# Molecular identification of the Danzhou chicken breed in China using DNA barcoding

**DOI:** 10.1080/23802359.2019.1638321

**Published:** 2019-07-13

**Authors:** Wenchuan Peng, Hui Yang, Keqi Cai, Lu Zhou, Zhen Tan, Kebang Wu

**Affiliations:** Laboratory of Tropical Animal Breeding, Reproduction and Nutrition, College of Animal Science and Technology, Institute of Tropical Agriculture and Forestry, Hainan University, Haikou, China

**Keywords:** Molecular identification, DNA barcode, cytochrome C oxidase subunit I (COI), Danzhou chickens

## Abstract

Mitochondrial cytochrome C oxidase subunit I (COI) has been used as a DNA barcode to identify population genetic diversity and distinguish animal species as it is variable enough to distinguish between species, yet suitably conserved. A new native chicken breed, named Danzhou chicken was discovered in Hainan, China in 2014, although identification is difficult by morphological examination alone. The mitochondrial COI genes of six chicken breeds, including four local and two imported breeds (Danzhou, Wenchang, Bawang, Beijing-You, Hy-Line Brown, and Ross) were compared and assessed in terms of their efficacy for DNA barcoding. The results showed that the number of COI gene variants in Danzhou chickens was less than those of other breeds, except Bawang and the genetic structure was relatively stable. The Kimura 2-parameter genetic distance between Danzhou chickens and the five other breeds was from ∼0.001 to 0.734. The genetic distance of the six breeds was ∼0.001–0.339, with that of Danzhou being the highest (0.339). Danzhou chickens clustered with Bawang and Wenchang chickens in the phylogenetic tree due to geographic closeness. Danzhou chickens could be identified more accurately using COI barcoding. Multiple molecular markers combined with morphological differences were more persuasive for identifying species.

## Introduction

Danzhou chicken is a small type of local variety in Hainan island of China, a good genetic resource in Hainan. Danzhou chickens have a series of characteristics and advantages, such as strong adaptability, disease resistance, crude feeding resistance, strong wildness, low-fat content, and delicious taste. They have the potential to develop into a high-quality chicken breed. The body weight, body size, living habits, and carcass quality of Danzhou chickens has been previously studied. However, Danzhou chickens cannot be accurately identified by morphological characters alone. Molecular methods for species identification are generally considered to be more accurate and efficient. Therefore, we aimed to determine an effective molecular-level method to identify Danzhou chickens.

DNA barcoding technology has been used for identification and classification of different taxa and has successfully identified new species and varieties (Hebert et al. 2004; Hajibabaei et al. 2006; Lane et al. 2007). Mitochondrial DNA sequences, including mitochondrial cytochrome oxidase I (COI), have many advantages as molecular markers. They are variable but sufficiently conserved, of appropriate sequence length, general primers for amplification and sequencing are available, molecular markers of COI can quickly identify species as a DNA barcode, and have been widely used in vertebrates and invertebrates classification, species identification, genetic diversity, and molecular evolutionary studies (Liu et al. 2001; Hebert et al. 2004; Donald et al. 2005; Wood et al. 2007; Ståhls and Savolainen 2008; Zhen et al. 2015; Wang et al. 2017; Tan et al. 2018). Hebert et al. (2003) first proposed to use a specific segment of the first half of the COI gene as a DNA barcode (Hebert et al. 2003). DNA barcodes can effectively differentiate 98% of the marine fish from freshwater fish (Ward et al. 2005).

A DNA barcode based on the COI gene has been used for species identification in chicken varieties (Yap et al. 2010; Yacoub et al. 2015), including the introduced and Chinese breeds that have a certain phylogenetic distance (Gao et al. 2007). In this study, Danzhou and five other chicken breeds were identified using the COI gene as a DNA barcode to evaluate the differential expression.

## Materials and methods

### Animal experiments and DNA extraction

A total of 315 Danzhou chickens, three local breeds, and two introduced breeds were used in the present study (Supplementary Table 1). Wenchang is the most typical local breed on Hainan Island. Bawang belongs to the red jungle fowl, Hainan subspecies. Beijing-You is a representative chicken breed in China. Ross is a commercial egg-laying breed from the UK. Hy-Line Brown is a well-known egg-laying breed from the USA. In this study, blood samples from each adult were collected from farms in collection localities Supplementary Table 1, cryopreserved, and transported to the laboratory for analysis. Then DNA was extracted from by TIANGEN blood DNA kit following the manufacturer’s instructions and the molecular markers were evaluated by DNA barcode technology (Hebert et al. 2004).

### Chicken cytochrome oxidase PCR amplification

The primers used in the experiment were designed according to the COI gene sequence of Chinese red raw chicken Cox1 (AP003322) published in GenBank: forward: 5′-GCACAGGATGGACAGTTTAC-3′, reverse: 5′-ATAGCATAGGGGGGTCTCAT-3′. The primer was synthesized by Shanghai Bioengineering Co., Ltd. (Shanghai, China). The PCR product was 651 bp in length. The PCR reaction system contained about 50 ng genomic DNA, 2 ml primers (20 molL^−1^), 2 ml dNTP mixture (2.5 mmol L^−1^), 16.75 ml d^2^H_2_O and 0.5 ml PFU enzyme (Shanghai Bioengineering Company). PCR products were analyzed by electrophoresis on 2% agarose gels. Then, PCR products were sent to Shanghai Bioengineering Co. Ltd. for two-way sequencing.

### Comparative analysis of chicken COI gene sequences

The sequences were read by Chromas Software, checked and proofread manually and corrected by bidirectional sequencing. The obtained sequences were compared with DNA MAN5.2.2 software (Lynnon Biosoft., USA) and the DNA barcode sequence of red raw chicken mitochondrial COI gene (AP003322) in GenBank was used as the standard for data analysis. The genetic distance was calculated by Kimura 2-parameter method in MEGA5.0 software and then the phylogenetic tree was constructed using the neighbor-joining method (Kumar et al. 2008).

## Results

### Gel electrophoresis of DNA and PCR products

The blood DNA molecular weight of the six breeds of chickens was about 650 bp (Supplementary Figure 1) and verified by real-time PCR that the molecular weight of each breed chicken as about 650 bp (Supplementary Figure 2) proved that DNA was successfully extracted and could be sequenced and followed up.

### Specific sites of the COI sequences of the six chicken breeds

There were 105 single nucleotide polymorphisms on the COI gene sequences and the variation points of the six chicken breeds were quite different (Supplementary Table 2). Among them, Danzhou had the largest number of variation points (36). The number of variation points in Bawang was the least (6).

### Analysis of genetic distance between populations of the six chicken breeds

Kimura’s 2-parameter genetic distance and the net genetic distance of COI gene sequences between Danzhou and the five other breeds is shown in Table 1 and ranged from ∼0.001 to 0.339. The genetic distance of Wenchang was 0. The genetic distance of Danzhou was 0.339. The genetic distances of the other four varieties were all small, within the range of ∼0.001–0.003. The intrabreed genetic distances were ∼0.001–0.734. The genetic distance between the two foreign breeds and domestic breeds was relatively large. Apart from Danzhou chickens, the genetic distances between the other three breeds and the two foreign species were large (all >0.7). This was probably due to hybridization and geographical origin. The genetic distance of the two foreign breeds was also large and the genetic distance of Hy-Line Brown was from ∼0.002 to 0.727; that of Ross was from ∼0.002 to 0.730. The net genetic distance of the six breeds was from ∼0.001 to 0.459.

**Table 1.** Kimura 2-parameter distance and net genetic distance (Da) between six chicken breeds.

Breed	Intrabreed	Danzhou	Bawang	Hy-Line Brown	Luosi	Beijing-You	Wenchang
Danzhou	0.339		0.153	0.306	0.307	0.153	0.153
Bawang	0.001	0.242		0.457	0.458	0.002	0.001
Hy-Line Brown	0.002	0.487	0.727		0.002	0.458	0.457
Luosi	0.003	0.488	0.730	0.002		0.459	0.458
Beijing-You	0.001	0.244	0.002	0.731	0.734		0.001
Wenchang	0.000	0.242	0.001	0.728	0.731	0.001	

Note: the upper triangle is the net genetic distance (Da) and the lower triangle is Kimura 2-parameter genetic distance.

### Phylogenetic tree of Danzhou and five other breeds of chicken

**Figure 1.**
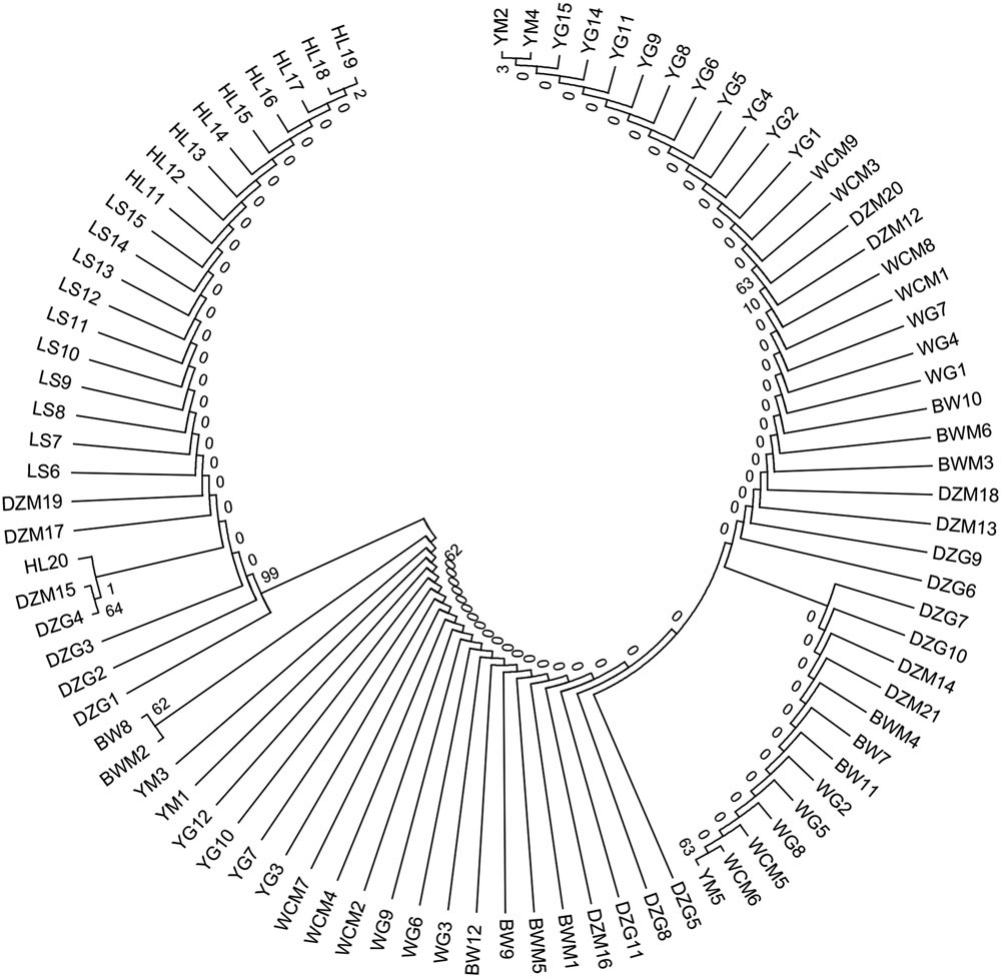
Phylogenetic tree of COI gene fragments of Danzhou and five other chicken breeds based on Kimura 2-parameter distance method.

The hybridization of Danzhou chicken was obvious in the phylogenetic tree (Figure 1). Some sequences were clustered with Bawang and Wenchang and the reference sequence from NCBI. The other sequences were clustered with Hy-Line Brown. Beijing-You, Wenchang, Bawang, Hy-Line Brown, and Ross were generally clustered on their respective branches.

## Discussion

Morphological characteristics and DNA barcoding are two of the main methods used for species identification. The most commonly used molecular markers for DNA barcoding are COI genes, which are located in the mitochondrial genome (mtDNA). The COI gene has been used for identification of different vertebrates and invertebrates (Tautz et al. 2002; Hebert et al. 2003; Kress et al. 2005; Ward et al. 2005; Zhen et al. 2015; Wang et al. 2017; Tan et al. 2018), including chickens (Gao et al. 2007).

Danzhou chickens are of small size, have a ‘beard’, yellow feet, and hemp feather. There are morphological differences between Danzhou chickens and the other breeds, yet difficult to distinguish from others accurately. Danzhou chicken population could be evaluated more comprehensively by changing the information of the heterotopic points. In this way, can we objectively understand the specific information and characteristics of Danzhou chickens.

This study measured six breeds of chicken, Danzhou, Wenchang, Bawang, Beijing-You, Hy-Line Brown, and Ross, and a total of 315 individual COI gene sequences, which were found to have a 651 bp variable region, 105 mutation loci. The main types of variation were inversion and transformation. Danzhou chickens had specific variations that provided more reliable information for evaluating Danzhou chickens at the molecular level. Therefore, DNA barcoding could effectively identify and distinguish chicken breeds and strains. These results were consistent with those of a previous study (Bondoc et al. 2015).

Danzhou chickens had a relatively low mutation rate and low genetic diversity, but the largest genetic distance (0.339). This was not entirely related to the formation of stable chicken breeds in Danzhou, which might be due to the lack of selective breeding in Danzhou chickens, consistent with the result of Yacoub et al. (Yacoub et al. 2015). The genetic distance of the other five breeds was relatively small indicating that the selection intensity in these breeds was higher than that in Danzhou chickens. This was partly due to the initial breeding of Danzhou chickens. The genetic distance between Danzhou chickens and Bawang, Wenchang, and Beijing-You chickens was 0.242, 0.242, and 0.244, respectively. The genetic distance between Danzhou chickens and Ross and Hy-Line Brown chickens was 0.488 and 0.487, respectively. The genetic distance from the foreign breeds was larger than from the local breeds indicating that Danzhou chickens were the most estranged from the two introduced breeds and relatively closely related to the three domestic breeds.

The phylogenetic tree showed that Danzhou chickens clustered with Wenchang, Bawang, and Hy-Line Brown chickens. Danzhou chicken was a low-intensity variety. The COI gene isolated from Danzhou, Wenchang, and Bawang chickens had similar sequences and insertion locations, the result of many hybridization events or recent differentiation. Relationships between geographical distribution, Danzhou, Bawang, and Wenchang chickens are from the Hainan area and a close genetic relationship between these breeds. Also, Hainan island is a relatively closed natural environment. Under such circumstances, for Danzhou chicken, which has a relatively low selection intensity, it is inevitable that they have a certain relationship with the Bawang and Wenchang chickens in Hainan. However, there were a series of crossovers between Danzhou chickens and the foreign Hy-Line Brown breed since its introduction to Hainan. This suggests that there were genetic exchanges between Danzhou chickens and Wenchang, Bawang, and Hy-Line Brown chickens.

In general, Danzhou chickens are used for breeding. The crossover between local and foreign chicken breeds was expected. Firstly, the six chicken breeds in this study have a large feeding range in Hainan. Due to geographical proximity and frequent trade, different chicken breeds inevitably experience different degrees of hybridization. Secondly, the relative conservatism of the COI gene means that genetic differences between breeds are relatively small. To evaluate the breeding potential of Danzhou chickens and other breeds, it is necessary to determine differences in morphology, growth performance, and cytology between them. Molecular aspects provide a molecular basis for the selection and breeding of Danzhou chicken.

There may be gene exchanges between Danzhou chickens and Wenchang, Bawang, and Hy-Line Brown chickens in Hainan province. Hainan Island is a relatively closed natural environment; therefore, the close relationship between Danzhou, Bawang, and Wenchang chickens is understandable. Since Danzhou chickens have relatively weak choices, let alone live in a relatively closed natural environment. In addition, our results indicate that there may have been a crossover between Danzhou chickens and imported Hy-Line Brown chickens. This might be due to crossbreeding when the Hy-Line Brown breed was introduced to Hainan.

## Conclusions

In this study, specific sequences of the mitochondrial COI gene were used as DNA barcodes for different breeds of chicken. It was found that this method can preliminarily assess the differences between Danzhou chickens and other breeds. Moreover, Danzhou chickens were identified and evaluated at the molecular level after morphological studies, which played an important role in improving the breeding of Danzhou chickens and the results of the present study will contribute to research on Danzhou chicken breeding strategies in the future. According to the results of this study, the mitochondrial COI, a specific segment of a specific gene, is used as the basis for DNA barcoding, based on its sequence polymorphism, specific site, and specific haplotype and sequence clustering results. Molecular identification of varieties has certain possibilities, also can be used for genetic diversity studies in local chickens, but standard DNA barcoding technology cannot effectively distinguish chicken breeds with small differences. DNA barcoding using the COI gene has the advantages of convenience, rapidity, economy, and accuracy in identifying different chicken breeds. However, the number of samples involved in this experiment and the sequences generated are not large enough. Therefore, further research is required on different breeds and the number of samples for sequencing needs to be expanded to verify the application of DNA barcoding.

## Supplementary Material

Supplemental Material

## References

[CIT0001] Bondoc OL, Gicana KRB, Hurtada JMUPA 2015 Analysis of genetic diversity and distances of some dog (*Canis lupus familiaris*) breeds based on DNA barcodes. Philipp J Vet Animal Sci. 40:1–12.

[CIT0002] Donald KM, Kennedy M, Spencer HG 2005 The phylogeny and taxonomy of austral monodontine topshells (Mollusca: Gastropoda: Trochidae), inferred from DNA sequences. Mol Phylogenet Evol. 37:474–483. 15936215 10.1016/j.ympev.2005.04.011

[CIT0003] Gao Y, Tu Y, Tong H, Wang K, Chen K, Gu R 2007 DNA barcoding application of mtDNA CO I gene in identifying six indigenous chicken breeds in China. J Agric Biotechnol.

[CIT0004] Hajibabaei M, Janzen DH, Burns JM, Hallwachs W, Hebert PD 2006 DNA barcodes distinguish species of tropical Lepidoptera. Proc Natl Acad Sci USA. 103:968–971. 16418261 10.1073/pnas.0510466103 PMC1327734

[CIT0005] Hebert PD, Penton EH, Burns JM, Janzen DH, Hallwachs W 2004 Ten species in one: DNA barcoding reveals cryptic species in the neotropical skipper butterfly *Astraptes fulgerator*. Proc Natl Acad Sci USA. 101:14812–14817. 15465915 10.1073/pnas.0406166101 PMC522015

[CIT0006] Hebert PDN, Cywinska A, Ball SL, deWaard JR 2003 Biological identifications through DNA barcodes. Proc Biol Sci. 270:313–321. 12614582 10.1098/rspb.2002.2218 PMC1691236

[CIT0007] Kress WJ, Wurdack KJ, Zimmer EA, Weigt LA, Janzen DH 2005 Use of DNA barcodes to identify flowering plants. Proc Natl Acad Sci USA. 102:8369–8374. 15928076 10.1073/pnas.0503123102 PMC1142120

[CIT0008] Kumar S, Nei M, Dudley J, Tamura K 2008 MEGA: a biologist-centric software for evolutionary analysis of DNA and protein sequences. Brief Bioinform. 9:299–306. 18417537 10.1093/bib/bbn017 PMC2562624

[CIT0009] Lane CE, Lindstrom SC, Saunders GW 2007 A molecular assessment of northeast Pacific Alaria species (Laminariales, Phaeophyceae) with reference to the utility of DNA barcoding. Mol Phylogenet Evol. 44:634–648. 17544704 10.1016/j.ympev.2007.03.016

[CIT0010] Liu LH, Xiao WH, Liu WW 2001 Effect of 5-Aza-2'-deoxycytidine on the P16 tumor suppressor gene in hepatocellular carcinoma cell line HepG2. World J Gastroenterol. 7:131–135. 11819749 10.3748/wjg.v7.i1.131 PMC4688690

[CIT0011] Ståhls G, Savolainen E 2008 MtDNA COI barcodes reveal cryptic diversity in the *Baetis vernus* group (Ephemeroptera, Baetidae). Mol Phylogenet Evol. 46:82–87. 18023596 10.1016/j.ympev.2007.09.009

[CIT0012] Tan L, Wang AB, Zheng SQ, Zhang XL, Huang CJ, Liu W 2018 Molecular characterization and phylogenetic analysis of *Taenia multiceps* from China. Acta Parasitol. 63:721–727. 30367774 10.1515/ap-2018-0085

[CIT0013] Tautz D, Arctander P, Minelli A, Thomas RH, Vogler AP 2002 DNA points the way ahead in taxonomy. Nature. 418:479. 10.1038/418479a 12152050

[CIT0014] Wang X, Kong L, Chen J, Matsukuma A, Li Q 2017 Phylogeography of bivalve *Meretrix petechialis* in the Northwestern Pacific indicated by mitochondrial and nuclear DNA data. PLoS One. 12:e0183221–e0183221. 28813498 10.1371/journal.pone.0183221 PMC5558932

[CIT0015] Ward RD, Zemlak TS, Innes BH, Last PR, Hebert PD 2005 DNA barcoding Australia's fish species. Philos Trans R Soc Lond B Biol Sci. 360:1847–1857. 16214743 10.1098/rstb.2005.1716 PMC1609232

[CIT0016] Wood AR, Apte S, MacAvoy ES, Gardner JP 2007 A molecular phylogeny of the marine mussel genus *Perna* (Bivalvia: Mytilidae) based on nuclear (ITS1&2) and mitochondrial (COI) DNA sequences. Mol Phylogenet Evol. 44:685–698. 17292632 10.1016/j.ympev.2006.12.019

[CIT0017] Yacoub HA, Fathi MM, Sadek MA 2015 Using cytochrome b gene of mtDNA as a DNA barcoding marker in chicken strains. Mitochondr DNA. 26:217–223. 10.3109/19401736.2013.825771 24020964

[CIT0018] Yap FC, Yan YJ, Loon KT, Zhen JLN, Kamau NW, Kumaran JV 2010 Phylogenetic analysis of different breeds of domestic chickens in selected area of Peninsular Malaysia inferred from partial cytochrome B gene information and RAPD markers. Animal Biotechnol. 21:226–240. 10.1080/10495398.2010.506334 20967642

[CIT0019] Zhen X, Wu B, Wang J, Lu C, Gao H, Qiao J 2015 Increased incidence of mitochondrial cytochrome C oxidase 1 gene mutations in patients with primary ovarian insufficiency. PLoS One. 10:e0132610. 26225554 10.1371/journal.pone.0132610 PMC4520565

