# Supplementary material for: Molecular identification of the Danzhou chicken breed in China using DNA barcoding
Source: Mitochondrial DNA B Resour. 2019 Jul 13;4(2):2459–63. doi: 10.1080/23802359.2019.1638321 (PMC7707848; doi:10.1080/23802359.2019.1638321)
Supplement: Supplemental Material [file TMDN_A_1638321_SM7567.zip › Supplementary File.docx]

**Supplementary File**

**Molecular identification of the Danzhou chicken breed in China using DNA barcoding**

Wenchuan Peng^1^, Hui Yang^1^, Keqi Cai^1^, Lu Zhou^1^, Zhen Tan^1*^, Kebang Wu^1*^

^1^ Laboratory of Tropical Animal Breeding, Reproduction and Nutrition, College of Animal Science and Technology, Institute of Tropical Agriculture and Forestry, Hainan University. Haikou 570228, China.

*Corresponding authors:

ZT, tankoer@qq.com

KW, wukebang66@sina.com

Supplementary Table 1 Experimental breed information

| Breed | Place of origin | Number of samples | Collection locality |
| --- | --- | --- | --- |
| Danzhou | Danzhou, Hainan | 63 | Xiuying farm, Hainan University |
| Wenchang | Wenchang, Hainan | 54 | Farms of Hainan Academy of Agricultural Sciences |
| Bawang | Changjiang County, Hainan | 36 | Hainan province Changjiang County, Wang Ling Ling Wang pheasant breeding base |
| Beijing-You | Beijing | 57 | Hainan Academy of Agricultural Sciences |
| Luosi | UK | 45 | Xiuying farm, Hainan University |
| Hy-Line Brown | USA | 60 | Xiuying farm, Hainan University |

Supplementary Table 2 COI gene sequence variation sites for the six chicken breeds

| Varieties | Danzhou | Bawang | Wenchang | Luosi | Hy-Line Brown | Beijing- You |
| --- | --- | --- | --- | --- | --- | --- |
| Number of samples | 63 | 36 | 54 | 45 | 60 | 57 |
| Mutation sites | 36 | 6 | 9 | 18 | 24 | 12 |


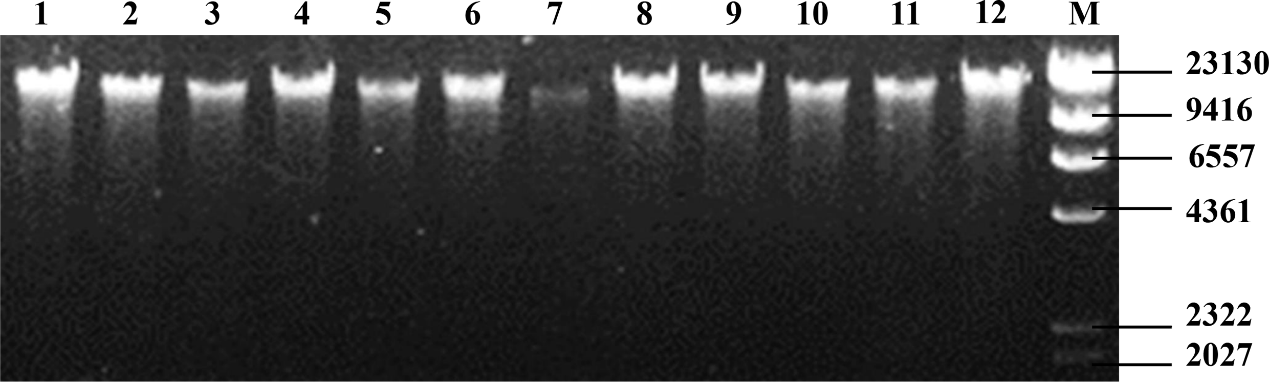


Supplementary Figure 1 DNA gel electrophoresis pattern of chicken breeds

1, 2 represent Danzhou chickens; 3, 4 represent Bawang chickens; 5, 6 represent Wenchang chickens; 7, 8 represent Beijing-You chickens; 9, 10 represent Hy-Line Brown chickens; 11, 12 represent Luosi chickens; M represent λDNA/Hind III marker.


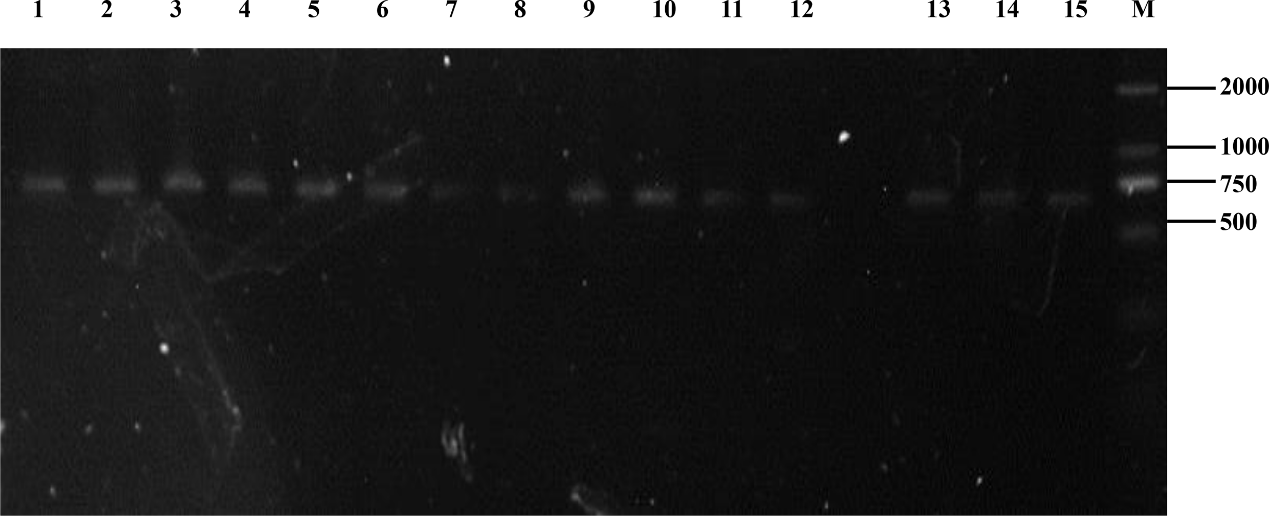


Supplementary Figure 2 Gel electrophoresis pattern of PCR products of chicken breeds

1, 2 represent Luosi chicken; 3, 4 represent Bawang chickens; 5, 6 represent Wenchang chickens; 7, 8 represent Beijing-You chickens; 9, 10 represent Hy-Line Brown chickens; 11–15 represent Danzhou chickens; M represents D2000 DNA marker.
